# Glutathione in Ovarian Cancer: A Double-Edged Sword

**DOI:** 10.3390/ijms19071882

**Published:** 2018-06-26

**Authors:** Sofia C. Nunes, Jacinta Serpa

**Affiliations:** 1 Centro de Estudos de Doenças Crónicas (CEDOC), NOVA Medical School/Faculdade de Ciências Médicas, Universidade Nova de Lisboa, Campo Mártires da Pátria 130, 1169-056 Lisboa, Portugal; sofia.nunes@nms.unl.pt; 2 Unidade de Investigação em Patobiologia Molecular do Instituto Português de Oncologia de Lisboa Francisco Gentil (IPOLFG), Rua Prof. Lima Basto, 1099-023 Lisboa, Portugal

**Keywords:** ovarian cancer, cancer metabolism, glutathione, cysteine, chemoresistance, platinum based drugs

## Abstract

Glutathione (GSH) has several roles in a cell, such as a reactive oxygen species (ROS) scavenger, an intervenient in xenobiotics metabolism and a reservoir of cysteine. All of these activities are important in the maintenance of normal cells homeostasis but can also constitute an advantage for cancer cells, allowing disease progression and resistance to therapy. Ovarian cancer is the major cause of death from gynaecologic disease and the second most common gynaecologic malignancy worldwide. In over 50 years, the overall survival of patients diagnosed with epithelial ovarian cancer has not changed, regardless of the efforts concerning early detection, radical surgery and new therapeutic approaches. Late diagnosis and resistance to therapy are the main causes of this outcome, and GSH is profoundly associated with chemoresistance to platinum salts, which, together with taxane-based chemotherapy and surgery, are the main therapy strategies in ovarian cancer treatment. Herein, we present some insights into the role of GSH in the poor prognosis of ovarian cancer, and also point out how some strategies underlying the dependence of ovarian cancer cells on GSH can be further used to improve the effectiveness of therapy.

## 1. Introduction

In 1888, Rey-Pailhade described philothion, a molecule that reacted spontaneously with sulphur, producing hydrogen sulphide [1,2]. Years later, Hopkins concluded that philothion and glutathione (GSH) were identical and, after several studies, in 1935, its structure was established as a tripeptide, composed of glutamic acid, cysteine and glycine [1,3]. Since then, countless papers have been published regarding GSH’s role in health and disease. 

GSH is found in Gram-negative bacteria and eukaryotes, being rare in Gram-positive bacteria. In mainly Archaea or amitochondrial eukaryotes, GSH is not generally found, leading Copley and Dhillon to suggest that bacteria transferred the genes for GSH biosynthesis to eukaryotes via the progenitor of mitochondria [4]. However, the evolutionary history of GSH biosynthesis genes is more complex. Data suggested that these genes evolved separately and their spread possibly involved events of horizontal gene transfer, including a transfer from an alpha-proteobacterium to a plant and also convergent evolution [4], as bacterial and eukaryotic proteins share a common structural fold but have very divergent sequences. Glutamic acid, cysteine and glycine are the building blocks of GSH, whose biosynthesis involves two ATP-requiring enzymes: glutamate cysteine ligase (GCL) and GSH synthetase. The first enzyme catalyses the formation of a dipeptide bond between the γ-carboxylate of glutamic acid and the amino group of cysteine and the latter catalyses the subsequent formation of a peptide bond between the cysteinyl carboxylate of γ-Glu–Cys and the amino group of glycine. Besides substrate availability, GCL is the rate-limiting enzyme in GSH synthesis, which presents two different subunits: the catalytic subunit (GCLC), containing the active site responsible for the bond formation between the amino group of cysteine and the γ-carboxyl group of glutamic acid, and the modifier subunit (GCLM) that interacts with GCLC, increasing the catalytic efficiency of GCLC [5]. GSH synthesis occurs in the cells cytosol. In theory, all cells are capable of synthesizing GSH; however, the liver present the highest capacity for its efflux, transporting GSH to the plasma and inter- and intra-organs [6]. Its degradation occurs extracellularly and it is catalysed by γ-Glutamyl transpeptidase and dipepdidase. The first enzyme leads to cysteineglycine production, which is subsequently cleaved by dipeptidase, producing cysteine and glycine [7].

Regarding GSH functions, Estrela [8] exhaustively described its impact on cell biology:

“GSH acts as a reducing agent and an antioxidant, is involved in the metabolism of xenobiotics and different cell molecules, is a free-radical scavenger, has a role in cell-cycle regulation and microtubular-related mechanisms, is a physiological reservoir of Cys, regulates Ca^2+^ homeostasis, regulates protein function and gene expression via thiol-disulphide exchange reactions, modulates lymphocyte functions and immune responses, and participates in the mitochondrial mechanisms that link opening of the permeability transition pore complex and activation of cell death” [8].

So, it is well known that GSH not only plays a main role in intracellular redox balance [3] but is also pivotal in cellular processes such as cell differentiation, proliferation and apoptosis [8,9,10]. Moreover, GSH was associated with resistance to ionizing radiation and drug-induced cytotoxicity [8,10,11,12,13]. However, radiotherapy is not commonly used to treat ovarian cancer.

As it plays so many important roles in cell biology, the linkage of GSH deregulation with disease is obvious. In fact, GSH metabolism impairment has been associated with several diseases such as cancer, kwashiorkor, seizure, Alzheimer’s and Parkinson’s disease, liver disease, cystic fibrosis, sickle cell anaemia, acquired immunodeficiency disease syndrome (AIDS), heart attack, stroke, diabetes [8,14,15], several mitochondrial disorders [16], Niemann Pick type C disease [17] and progressive multiple sclerosis [18,19]. 

Here we will review the role of GSH in cancer, focusing on ovarian cancer, the major cause of death from gynaecologic disease and the second most common gynaecologic malignancy worldwide [20,21]. This molecule is profoundly associated with platinum salt resistance, one of the main treatment strategies for ovarian cancer, and is thus of extreme importance as a potential threat to ovarian cancer cells.

## 2. Ovarian Cancer—An Overview

Ovarian cancer is the major cause of death from gynaecologic disease and the second most common gynaecologic malignancy worldwide [20,21], especially due to late diagnosis and resistance to therapy [22]. Epithelial ovarian cancer (EOC) includes most malignant ovarian neoplasms [23] and is composed of different diseases that can be classified based on morphologic and molecular genetic features: serous (OSC; low and high grade), endometrioid (EC), clear cell (OCCC) and mucinous (MC) carcinomas. Each histological subtype was already associated with a specific genetic and transcriptional signature: low-grade OSC generally comprising BRAF, KRAS, NRAS, Erb-B2 Receptor Tyrosine Kinase 2 (ERBB2) mutations; high-grade OSC comprising mutations in Tumour Protein P53 (TP53), BRCA1/2, Neurofibromin 1 (NF1), RB Transcriptional Corepressor 1 (RB1), Cyclin Dependent Kinase 12 (CDK12), homologous recombination repair of DNA damage defective in approximately 50% of high grade serous cancers and alterations in signalling pathways such as PI3/Ras/Notch/ FoxM1. EC subtypes involve mutations in AT-Rich Interaction Domain 1A (ARID1A), Phosphatidylinositol-4,5-Bisphosphate 3-Kinase Catalytic Subunit Alpha (PI3KCA), Phosphatase And Tensin Homolog (PTEN), Protein Phosphatase 2 Scaffold Subunit Alpha (PPP2R1α), and mismatch repair deficiency; OCCC subtype comprises de novo expression of HNF1β [24,25] and ARID1A, PI3KCA, PTEN, Catenin Beta 1 (CTNNB1) and PPP2R1α mutations; MC comprises tumours with mutations in KRAS and high frequency of ERBB2 amplification with overexpression of mucin-coding genes [26,27].

**Figure 1:**
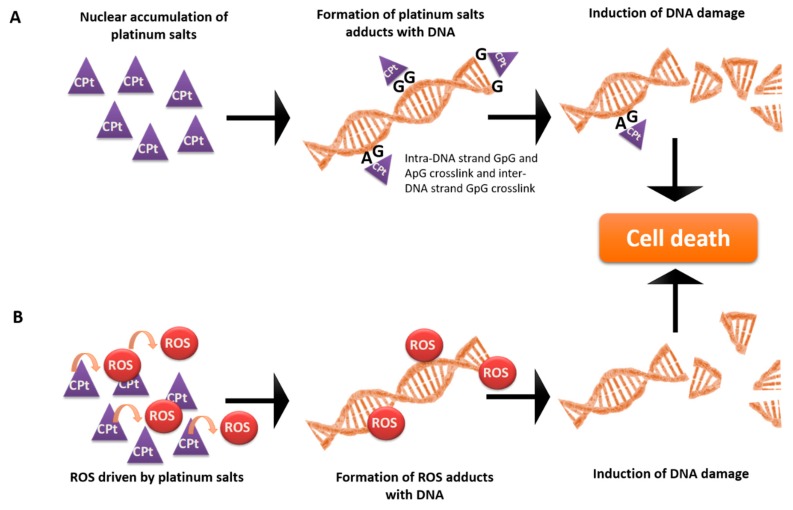
Mechanism of action of platinum salts. Platinum salts cause cancer cells’ death through two mechanisms: (**A**) direct covalent bonds (adducts) with DNA helix, provoking DNA breaks, and (**B**) the generation of reactive oxygen species (ROS) through chemical interaction with the organic components of the cell, namely proteins. Despite disturbing the metabolic equilibrium of the cell, these ROS will establish adducts with DNA, inducing DNA damage. Based on Benhar et al. [37] and Chaney et al. [38].

OSC is the most prevalent histological type [22], with diagnosis at an advanced stage in approximately 70% of patients [28]. In contrast, OCCC is a rather uncommon histological type of ovarian cancer that is frequently diagnosed at an initial stage [29]. However, OCCC tumours present markedly different clinical behaviours compared to other EOC subtypes, presenting, generally, a poor prognosis given the intrinsic chemoresistance to conventional platinum, or taxane-based therapy [29], which, together with surgery, constitute the standard care for ovarian cancer [30]. Importantly, therapy based on platinum salts involves ROS-mediated apoptosis [31,32,33]. Cisplatin and carboplatin are highly reactive molecules that bind to RNA, DNA and proteins, leading to the formation of adducts [30,33]. The nuclear DNA adducts are thought to be mainly responsible for cisplatin cytotoxicity (Figure 1), inducing cell death as a result of DNA damage and inhibition of replication and transcription [30,33]. Marullo et al. reported a role of cisplatin also in ROS production (Figure 1) driven by protein synthesis impairment [33]. Paclitaxel cytotoxicity is associated with its binding to intracellular β-tubulin, which leads to microtubule stabilization, G2-M arrest and apoptosis [30,34]. However, Alexandre et al. also reported that Paclitaxel exposure induces ROS production, as H_2_O_2_ accumulation showed to be an early and crucial step in paclitaxel cytotoxicity [32].

Despite initial response to treatment, 85% of advanced ovarian cancer patients suffer from disease recurrence [35]. Moreover, in over 50 years, the overall survival of patients diagnosed with epithelial ovarian cancer has not changed, regardless of efforts aimed at early detection, radical surgery and new therapeutic approaches [36]. Therefore, we will focus on the role of GSH in mediating resistance to the pro-oxidative chemotherapy used in ovarian cancer.

## 3. GSH-Mediated Platinum-Based and Taxane-Based Chemoresistance in Ovarian Cancer

Cisplatin and carboplatin are platinum analogues in which carboplatin presents a similar efficacy to cisplatin, but with significantly less toxicity [30,39]. GSH is known to mediate resistance to both cisplatin and carboplatin through several mechanisms such as drug uptake reduction and increased intracellular drug detoxification/ inactivation (Figure 1), increased DNA repair and inhibition of apoptosis drug-induced oxidative stress [40,41,42,43]. Specifically in ovarian cancer, although with some controversy [44,45], several reports already associated high GSH levels or glutathione S-transferase P1 (GSTP1) activity with cisplatin or carboplatin resistance [46,47,48,49,50,51,52,53]. More recently, Sawers et al. [54] have shown that the stable deletion of GSTP1 significantly and selectively increased sensitivity both to cisplatin and carboplatin in A2780 ovarian cancer cells [54]. Crawford and Weerapana reported a dichlorotriazine-containing compound (LAS17) that selectively and irreversibly inhibited GSTP1 activity, thus providing a promising cancer therapeutic target [55]. In addition, Chen et al. have shown that the overexpression of the microRNA miR-133b increased ovarian cancer cell sensitivity to both cisplatin and paclitaxel by decreasing glutathione GSTP and multidrug resistance protein 1 (MDR1) expression [56]. Importantly, Lopes-Coelho et al. [57] were able to find differences among ovarian cancer histotypes in terms of carboplatin resistance and GSH levels. They found that OCCC cells were more resistant to carboplatin than OSC cells, and that the inhibition of GSH production by BSO sensitized these cells to carboplatin, both in vitro and in vivo [57]. Those results highlight that ovarian cancer is a complex disease in which each histotype presents unique features in terms of thiol metabolism and response to chemotherapeutic agents that should be taken into account in the clinical context. 

Paradoxically, concerning the role of GSH in paclitaxel resistance, Liebmann et al. have shown that the depletion of cellular GSH by L-BSO resulted in increased resistance to taxol in MCF-7 and A549 cells [58]. However, Medeiros et al. reported that patients with ovarian cancer, who are carriers of glutathione-S-transferase class µ (*GSTM1)-null* genotype or carriers of non-*GSTM1-wt/GSTT1-wt* genotypes, when treated with both paclitaxel and cisplatin, present higher mean survival time [59], suggesting a role for GSH in paclitaxel resistance also. 

## 4. GSH Antagonists in Ovarian Cancer

Given the crucial role of GSH in ovarian cancer chemoresistance, efforts to antagonize its effects on cancer cells have already been developed. So far, different strategies have been reported, like inhibiting GSH biosynthesis, using GSH analogues or drugs targeting S-glutathionylation of proteins.

In the context of ovarian cancer, the effect of agents that lead to GSH depletion has already been addressed. Hong and colleagues have reported the effect of a phytochemical β-phenylethyl isothiocyanate (PEITC), on ROS accumulation and consequent UPR-mediated apoptosis in SKOV3 ovarian cancer cell line [60]. This drug is involved in cysteine modification of side chains in GST, leading to its irreversible inhibition. Importantly, this drug did not affect normal ovarian epithelial cells or peripheral blood mononuclear cells [60]. However, the authors also reported that the addition of *N*-acetyl-l-cysteine, a ROS scavenger, was able to revert the PEITC-induced cell death [60]. These results show that one possible mechanism of resistance to this drug would be the upregulation of alternative antioxidant responses capable of counteracting this ROS accumulation, such as thioredoxins. Similar findings of PEITC on apoptosis induction in OVCAR3 cells were also reported [61].

BSO was another drug involved in the inhibition of GSH production reported in the ovarian cancer context. BSO is a specific inhibitor of GCL, a critical enzyme in GSH biosynthesis. As already mentioned, Lopes-Coelho et al. have reported that the inhibition of GSH production by BSO sensitized OCCC cells to carboplatin exposure, both in vitro and in vivo [57]. Interestingly, in a mice model of breast cancer and in cell lines including lymphoma, glioblastoma, and non-small-cell lung carcinoma, Harris et al. reported that the GSH antioxidant pathway was required for cancer initiation but not for its progression due to the existence of alternative antioxidant pathways. They have shown that the depletion of GSH driven by BSO was able to prevent the malignant transformation but, at the disease onset, only the combined inhibition of GSH and thioredoxin antioxidant pathways led to synergistic cancer cell death, both in vitro and in vivo [62]. Those results support the idea that cancer cells might evolve resistance mechanisms in other antioxidant pathways in order to counteract oxidative stress, thus being able to survive. In a clinical context, BSO was shown to be a chemosensitizer but with no clinical advantages due to the severe adverse effects, thus clinical trials are no longer being developed [63].

Some studies have also tested the effect of natural compounds. Wang and colleagues explored the role of gossypol, a phenolic aldehyde present in cotton and tropical plants, in ovarian cancer cells and have shown that apoptosis was related to GSH depletion and changes of the thiol redox state in several proteins [64]. Importantly, they observed changes in redox-sensitive cysteine residues in proteins involved in metabolism homeostasis and stress responses [64].

Recently, 3-bromopyruvate (3-BP), known as an antagonist of lactate and pyruvate, was also found to form GSH conjugates, leading to its depletion and allowing chemoresistance reversion in several cancer types [65]. Gandham and colleagues, using SKOV-3 spheroids, showed 3-MP cytotoxicity in the ovarian cancer context [66], thus being a general anti-cancer strategy with promising results also in ovarian cancer [65].

Acetaminophen, known as paracetamol, was also reported to deplete GSH, contributing to chemoresistance reversion [65]. Using SKOV3, Wu and colleagues have demonstrated the benefits of acetaminophen as a co-adjuvant drug, improving cisplatin and paclitaxel efficacy [67]. They also reported the enhanced effect of cisplatin combined with this drug on reducing tumour recurrence in a SKOV3 subcutaneous xenograft mouse model [67]. Very recently, Lian et al. reported the long-lasting activation of paracetamol and its cytotoxic effects in SKOV3 cells using a tyrosinase-MOF nanoreactor, an enzymatic nanoreactor based on metal–organic frameworks (MOFs) [68]. This strategy may allow, simultaneously, the achievement of higher cancer cell selectivity with consequent decreased systemic toxicology and an increase in the half-lives of the activating enzymes, leading to lasting paracetamol activation [68].

GSH analogues were also reported in ovarian cancer. Telcyta (TLK286) is a GSH analogue that, when metabolized by GSTP1-1, releases a reactive tetrakis (chloroethyl) phosphorodiamidate fragment and a glutathione analogue vinyl sulphone that, following activation, triggers the stress response pathway, leading to cellular apoptosis induction [69]. Telcyta has been tested in Phase II and III clinical trials for ovarian cancer treatment, as a monotherapy regime or in combination with other chemotherapeutic agents [63]. However, a Phase III (NCT00350948) randomized study of Telcyta and doxorubicin in platinum refractory ovarian cancer patients (ASSIST-5) showed that Telcyta led to poorer outcomes than standard strategies [63]. Mechanisms such as increased/decreased rates of drug efflux/influx could explain the failure of those strategies.

Drugs that target S-glutathionylation—a reversible mechanism of protein regulation [70]—have already been developed in ovarian cancer. Anthracyclines, such as doxorubicin, induce cyclical redox reactions, leading to S-glutathionylation of cellular proteins [71]. However, according to a systematic review and meta-analysis of randomized trials in the ovarian cancer context, a liposome-encapsulated formulation of doxorubicin did not improve the overall survival of ovarian cancer patients when compared to other strategies in all phases of disease [72]. This study also reported a marginal advantage in progression-free survival—only in platinum-sensitive patients and as a second-line treatment [72]. Those results support the existence of a link between platinum-based therapy resistance and S-glutathionylation-induced resistance. 

NOV-002 consists of other inducer of S-glutathionylation tested in ovarian cancer. However, in chemotherapy-resistant ovarian cancer patients (NCT00345540), NOV-002 in combination with carboplatin failed to reach its aim, and thus was withdrawn [63].

The observed failure of drugs targeting S-glutathionylation in clinical trials regarding ovarian cancer might be explained by resistance mechanisms such as increased/decreased rates of drug efflux/influx as cellular redox status affects transporters’ function or resistance to apoptosis through caspase-3 S-glutathionylation [73].

## 5. The Paradox of Antioxidants as Co-Adjuvants of Pro-Oxidative Drugs 

So far, the studies concerning GSH-mediated resistance to conventional chemotherapy have led to the hypothesis that targeting redox balance via GSH deprivation in combination with pro-oxidant therapies should be effective in cancer treatment. However, antioxidant supplementation during chemotherapy was also rationalized as a way to counteract the deleterious effects of cancer-induced antioxidant depletion [74]. In fact, it has been suggested that antioxidants could have a beneficial effect as co-adjuvants of chemotherapy in patients with ovarian cancer. Di Re et al. [75] reported that GSH addiction is safe, well-tolerated and very effective in ovarian cancer patients with bulky disease subject to high-dose cisplatin administration [75]. Confirming this study, years later, Smyth and colleagues [76] demonstrated in a clinical trial that GSH addition together with cisplatin was less toxic and increased the quality of life of advanced-stage ovarian cancer patients [76]. However, in these studies, parameters such as the median survival of patients, tumour recurrence and tumour growth rate were not assessed; only quality of life parameters were assessed, so it is unclear if GSH addition is in fact safe. Ladas et al. reviewed the efficacy and safety of antioxidant use during cancer treatment and concluded that there is a lack of evidence that individual antioxidant vitamin supplements reduce the toxicity associated with anticancer therapies [74]. Probably the antioxidant intake during chemotherapy improves the quality of life of patients, because the adverse effects of cytotoxics are minimized, but the cytotoxic effectiveness on cancer cells is also reduced, compromising the overall efficacy of the oxidative therapy. 

The wide variation in extracellular (e.g., 2–20 µmol/L in plasma) and intracellular (0.5–10 mM) GSH concentrations in animal cells [15] led to the notion that this leads to a large difference in reducing potential between the intracellular and extracellular compartments that could be exploited for triggered intracellular delivery of several bioactive molecules [77]. Given that tumour tissues are highly reducing and hypoxic compared to normal tissues and present higher GSH concentrations [77,78], the reducible bioconjugates could be valuable for tumour-specific drug delivery [77], as it was described for GSH-responsive micelles that improves paclitaxel delivery [79,80,81,82,83]. This led to the development of a targeted intracellular delivery system of paclitaxel based on redox-sensitive conjugates of hyaluronic acid–deoxycholic acid, which was tested in human breast adenocarcinoma cell lines [84]. It was proven that cancer cells were able to uptake these micelles by endocytosis and, in tumour-bearing mice, redox-sensitive micelles presented a much higher tumour-targeting capacity than in the control mice treated with paclitaxel without micelles [84]. Yan et al. also reported a novel polymer–paclitaxel conjugate based on a disulphide linker with a high Paclitaxel loading amount capacity, using HEK293 and HeLa cells [80]. More recently, Pei et al. reported GSH-responsive nanovesicles that exhibited improved paclitaxel solubility and effective cellular uptake in HeLa and HepG2 cells [81]. As far as we know, in an ovarian cancer context, those micelles have not been tested yet, but we predict that the response of ovarian cancer cells to those micelles would differ between histological types, in which OCCC cells that are more dependent on thiol metabolism would be even more suitable for this delivery system. 

Taken together, there is not enough evidence to support the use of GSH as a co-adjuvant of chemotherapeutic drugs. However, evidence suggests that cancer cells’ dependence on GSH can be further exploited in ovarian cancer cells to trigger anti-cancer drug delivery to ovarian cancer cells, which could be a promising strategy in fighting ovarian cancer.

## 6. GSH as a Cysteine Reservoir

One of the most important functions of GSH is the storage of cysteine, given its high extracellular instability that rapidly auto-oxidizes to cystine (the oxidized dimer form of cysteine) [8].

**Figure 2:**
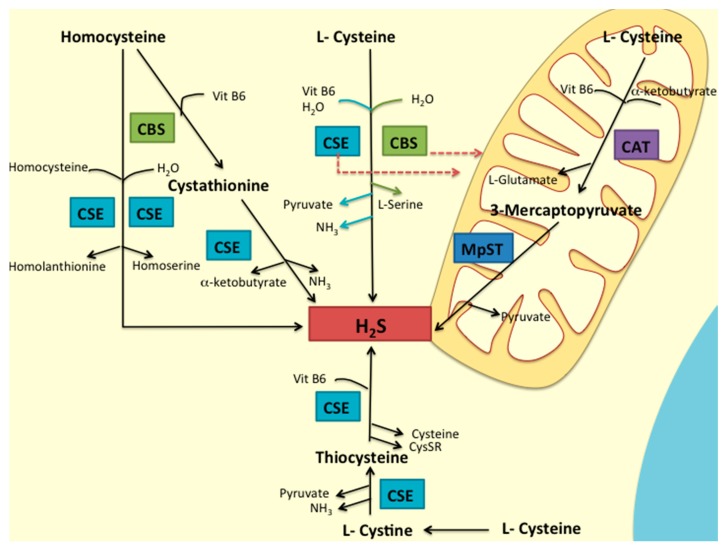
Pathways of H_2_S production in mammalian cells. H_2_S is synthesized from homocysteine or cysteine by the enzymes CBS and CSE, or by the sequential activity of CAT and MpST. In physiological conditions, CBS and CSE produce cytosolic H_2_S, whereas, in case of cellular stress, the enzymes can be relocated to the mitochondria, supporting CAT/MpST activities. Adapted from Wang [92] and Olson et al. [95].

The contribution of cysteine to cancer cells’ survival due to hydrogen sulphide (H_2_S) generation [85,86,87,88,89,90] and as a precursor of GSH [7,57,91] has been explored. In mammalian cells, H_2_S is mainly generated via enzymatic pathways and from the metabolism of L-cysteine by the catalysis of three key enzymes: cystathionine β-synthase (CBS), cystathionine γ-lyase (CSE) and by 3-mercapto-pyruvate sulfurtransferase (MpST), accompanied by cysteine aminotransferase (CAT) [92]. Besides MpST, in conjunction with CAT [93], CSE [94] and CBS [85,86] were also associated with H_2_S generation and mitochondrial ATP production (Figure 2).

In the context of ovarian cancer, Bhattacharyya et al. found a role of CBS in promoting ovarian tumour growth, drug resistance and cellular bioenergetics [85]. Recently, Cochrane and colleagues, through a proteomic screen, reported that CSE overexpression was consistent in OCCC and rare in endometrioid and high-grade serous ovarian cancer [96]. This fact suggests that the worse prognosis of OCCC could be due to a higher dependence of cancer cells on cysteine metabolism. Moreover, Poisson et al., through a metabolomics analysis, reported that platinum-sensitive and -resistant ovarian cancer cells presented significant differences involving cysteine and methionine metabolism [97]. They reported that 5-methylthioadenosine, cystathione, cysteine, cysteine sulphinic acid and methionine decreased in the resistant cells compared to the sensitive cells, while alanine, aspartate, reduced GSH, *S*-formyl-l-methionine and pyruvate were increased in resistant cells [97].

**Figure 3:**
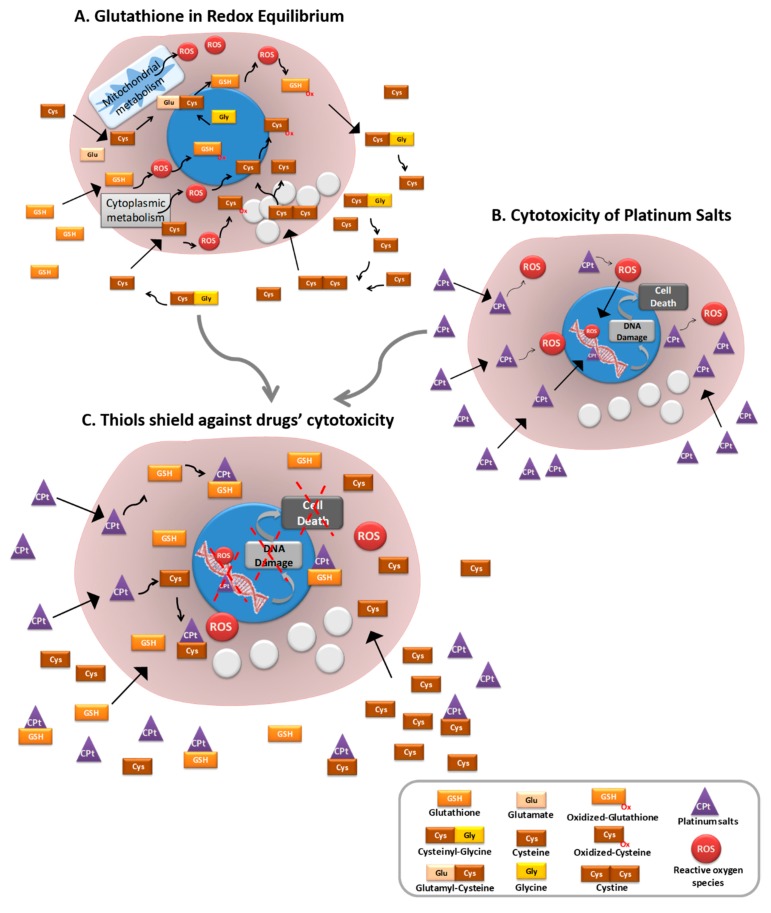
Gluthathione serves as a shield to protect ovarian cancer cells from conventional platinum drugs. (**A**) Glutathione is a pivotal player in the maintenance of redox equilibrium, which allows for homeostatic metabolic flux in a cell. Cysteine is the thiol component of glutathione and can also work as a ROS scavenger. (**B**) Platinum-salt-based drugs are commonly used to treat cancer; their cytotoxic mechanisms induce DNA damage, directly through covalent bonds (adducts) or through the generation of reactive oxygen species (ROS). (**C**) Cancer cells whose metabolism relies on thiols are better protected against platinum-salt-derived drugs. Glutathione and cysteine can bind to platinum salts, abrogating their cytotoxic effect and protecting cancer cells from DNA damage and consequently from death. Thiols allow for the regulation of several metabolic processes central to cancer cells, thus allowing cancer cells’ survival in a stressful and detrimental environment.

Okuno et al. [98] reported that the transport system x_c_^-^ (xCT), involved in cystine and glutamate transport, was associated with intracellular GSH level and with cisplatin resistance in human ovarian cancer cell lines. Wang and colleagues explored the role of ovarian cancer cells’ microenvironment and showed that fibroblasts decreased the nuclear accumulation of platinum in cancer cells through glutathione and cysteine release. On the other hand, they demonstrated that CD8^+^T cells counteracted this resistance by changing GSH and cystine metabolism in fibroblasts [99]. These results highlight the importance of cysteine in GSH-mediated resistance to cisplatin (Figure 3), as intracellular cysteine is a rate-limiting precursor for GSH synthesis [98]. Moreover, cysteine is the thiol component of GSH and the reversible thiolation of proteins was already associated with the regulation of several metabolic processes such as enzyme activity, transport activity, signal transduction and gene expression [10]. Several proteins—such as RAt Sarcoma vírus (Ras) protein, Jun N-terminal kinase (JNK)-2, Activator protein 1 (AP-1), Nuclear Factor-kappaB (NFkB), protein kinase C (PKC), caspase, thioredoxin and tumour protein 53 (p53) [10,100]—which are known to have important roles in ovarian cancer [101,102,103,104,105,106,107,108,109,110,111,112], are regulated by thiol oxidation [100]. Interestingly the oxidation of cys residues of p53 induces protein inactivation, which accounts for carcinogenesis [100]. Visscher et al. reported that many oncogenic mutations consist of an insertion of a novel cysteine in the protein sequence [113]. They also reported that acquired cysteines account for at least 12% of all activating mutations found in Kirsten ras oncogene (KRAS) in cancer, and 88% of mutations in fibroblast growth factor receptor (FGFR). They suggested that acquired cysteines often play a role in tumourigenesis [113], probably by contributing to tumour suppressor genes’ inactivation and oncogenes’ activation. 

Interestingly, Moheel and colleagues reported an active methylene quinuclidinone compound derived from the prodrug APR-246 (PRIMA-1^MET^), which not only binds to cysteine residues in mutant p53, restoring its wild-type conformation, but also binds to cysteine from GSH, leading to decreased intracellular free GSH concentrations [114]. This compound was able to restore the sensitivity to both cisplatin and doxorubicin of p53-mutant drug-resistant ovarian cancer cells [114]. 

Together, ovarian cancer cells’ dependence on GSH might be mainly due to its component cysteine, as it has a role in GSH-mediated and H_2_S-mediated chemoresistance, promotes redox balance and allows for the regulation of several metabolic processes central to cancer cells, thus permitting cancer cells’ survival in a stressful and detrimental environment (Figure 3).

Our studies on ovarian cancer metabolic adaptive features, accounting for cell fitness upon stressful conditions and chemoresistance, have shown that cysteine is beneficial to cancer cells. Hypoxia is a common condition in the cancer microenvironment, and it is well established that cancer cells that are capable of surviving hypoxia will be more prone to progression. We have shown that cysteine is a facilitator for cancer cells’ adaption to hypoxia and also to carboplatin, and for most ovarian cancer cell lines this adaptive capacity relies on CD133+ cells, putatively cancer stem cells [115,116].

With the exception of GSH, our data revealed increased thiol levels in peripheral blood from patients with ovarian tumours (benign and malignant) compared to healthy individuals. Interestingly, peripheral blood protein-*S*-cysteinylation levels were able to distinguish healthy donors from patients with ovarian neoplasms (benign and malignant) and total free cysteine levels distinguished benign tumours from malignant tumours [115], showing that cysteine levels are putative biomarkers for ovarian cancer’s early diagnosis. As late diagnosis is one of the most important barriers accounting for poor outcome and high mortality, our findings pave the path for an early diagnosis method. 

## 7. Conclusions

GSH plays many important roles in cell biology, and evidence suggests that cancer cells are especially dependent on it. Strategies that aim to deplete cellular GSH or target S-glutathionylation of proteins have failed so far. However, exploiting GSH to trigger the delivery of anti-cancer drugs to cancer cells is promising in ovarian cancer treatment. In addition, as GSH is an important cysteine storage, targeting this amino acid’s uptake by cancer cells could be another promising strategy to fight cancer, as cysteine allows for not only GSH-mediated but also H_2_S-mediated chemoresistance. Moreover, cysteine is the GSH component responsible for the reversible thiolation of proteins that impact the regulation of several metabolic processes that are central to cancer cells’ sustainability within the tumour microenvironment. Taken together, targeting GSH, especially through its component cysteine, could be a promising tool to fight ovarian or other types of cancer. Inhibitors of cysteine transporters like sulfasalazine are already used in the context of other diseases such as Crohn’s disease and rheumatoid arthritis. This review supports the concept of “teaching old drugs new tricks,” as this drug could also be a valuable tool in the context of ovarian cancer treatment.
